# Resolving Peak Overlap in HPLC Analysis of Glycerol Oxidation Products by Utilizing Various Detectors: Application to BiVO_4_ Photoanodes

**DOI:** 10.1021/acsomega.4c07497

**Published:** 2025-03-18

**Authors:** Heejung Kong, Siddharth Gupta, Matthew T. Mayer, Eva Ng, Camilo A. Mesa, Sixto Giménez, Fatwa F. Abdi, Roel van de Krol, Marco Favaro

**Affiliations:** † Institute for Solar Fuels, Helmholtz-Zentrum Berlin für Materialien und Energie GmbH, Hahn-Meitner-Platz 1, 14109 Berlin, Germany; ‡ Institute for Chemistry, Faculty II − Mathematics and Natural Sciences, Technische Universität Berlin, Straße des 17. Juni 124, 10623 Berlin, Germany; § Electrochemical Conversion, Helmholtz-Zentrum Berlin für Materialien und Energie GmbH, Hahn-Meitner-Platz 1, 14109 Berlin, Germany; ∥ Institute of Chemistry and Biochemistry, Department of Biology, Chemistry, and Pharmacy, Freie Universität Berlin, 14195 Berlin, Germany; ⊥ Institute of Advanced Materials, Universitat Jaume I, Avinguda de Vicent Sos Baynat, s/n, 12006 Castelló de la Plana, Spain; # School of Energy and Environment, City University of Hong Kong, 83 Tat Chee Avenue, Hong Kong SAR, China

## Abstract

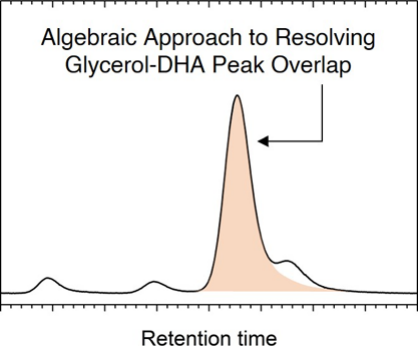

Glycerol, often considered a waste byproduct of biodiesel production, holds the potential for conversion into chemicals of varying economic value, such as dihydroxyacetone (DHA) and formic acid (FA). Hence, accurate identification and quantification of glycerol oxidation reaction (GOR) products are crucial for glycerol valorization research and practical deployment. High-performance liquid chromatography (HPLC) is the preferred analytical method for these purposes due to its proficiency in separating and quantifying components in liquid mixtures, even in the presence of diluted solutes. On the other hand, peak overlap in chromatograms, especially among glycerol, DHA, and FA, poses a notable challenge in the analysis of GOR products. This study introduces a quantification method aimed at resolving peak overlaps in HPLC analysis of GOR products. Initially, we examine the optical properties of glycerol and GOR products to identify optimal wavelengths for spectrophotometric HPLC analysis and detection. Subsequently, we propose an algebraic approach to resolve the peak overlap of glycerol, DHA, and FA using various detectors, including the refractive index detector (RID) and the variable wavelength detector (VWD). This method is applied to analyze the GOR products of undoped, nonco-catalyzed nanoporous BiVO_4_ photoanodes, which have shown an intrinsic catalytic activity toward GOR products in previous studies.

## Introduction

**Scheme 1:**
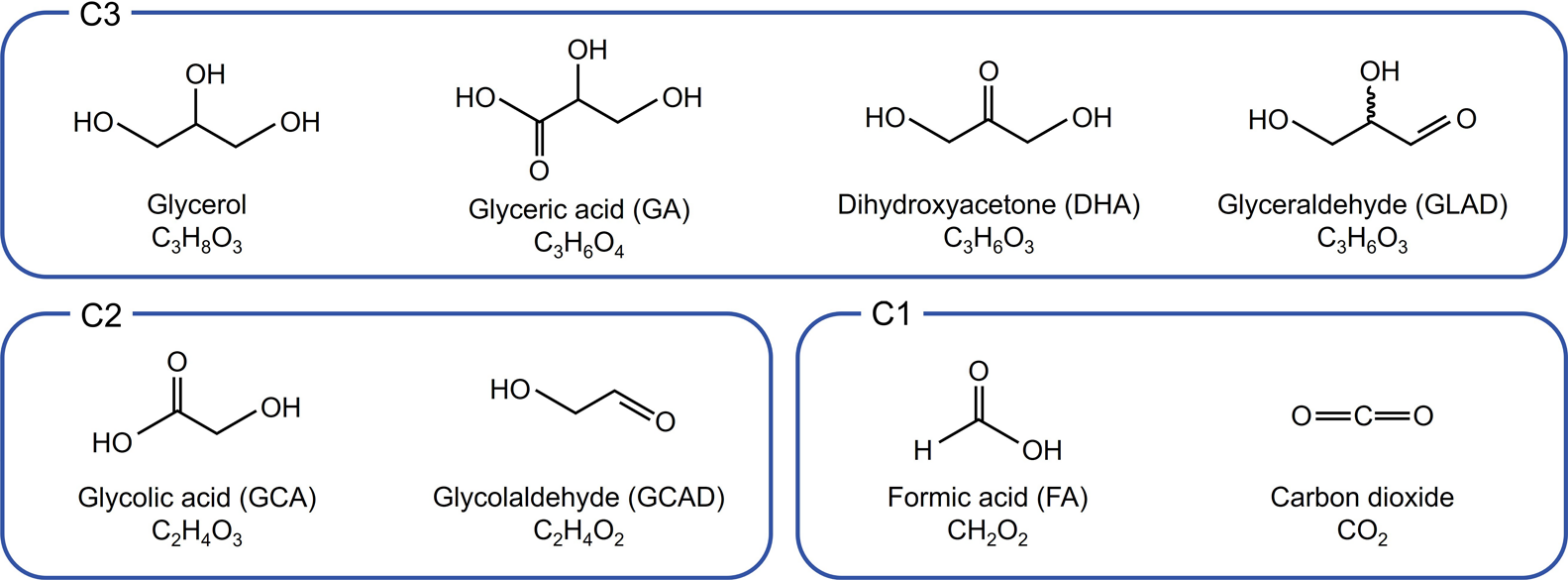
Glycerol and Various Glycerol Oxidation Reaction (GOR) Products

Biodiesel, a renewable and biodegradable fuel derived from nonedible plants, waste oils, and animal fats, offers a sustainable alternative to conventional diesel.^1^ Glycerol is produced as a byproduct of biodiesel production, with an approximate yield of 0.1 tons for every ton of biodiesel.^2,3^ This has led recently to an oversupply, rendering glycerol a low-value waste product. Nevertheless, glycerol holds the potential to be converted into more valuable chemicals, such as dihydroxyacetone (DHA), glycolaldehyde (GCAD), glyceraldehyde (GLAD), glyceric acid (GA), and glycolic acid (GCA), as illustrated in Scheme 1.^4,5^ Incorporating glycerol into a (photo)electrolyzer allows for the simultaneous production of glycerol oxidation reaction (GOR) products at the anode and hydrogen at the cathode.^6,7^ This integration can significantly improve the techno-economic feasibility of (photo)electrolysis cells.^8^ In this context, the identification and quantification of GOR products are crucial for research and industrialization efforts focused on glycerol valorization. This importance stems from the significant variations in the market prices of GOR products. For instance, the market price of formic acid (FA) is comparable to, or even lower than, that of refined glycerol, whereas the market prices of DHA, GCAD, and GLAD are several hundred times higher compared to that of glycerol.^9,10^

High-performance liquid chromatography (HPLC) is the predominant method for the analysis of GOR products, renowned for its capacity to efficiently separate, identify, and quantify components within a liquid mixture, even in the presence of diluted solutes. HPLC is capable of analyzing compounds soluble in any liquid that can act as the mobile phase.^11^ HPLC employs two main types of detectors: (1) solute property detectors, which are sensitive to specific solute characteristics, such as absorption within the UV–vis wavelength range, with variable wavelength detectors (VWDs) being a notable example, and (2) bulk property detectors, which monitor changes in one or more of the physical properties of both the mobile phase and the solute.^12^ The refractive index detector (RID) is a key member of this latter category. RIDs measure variations in the refractive index (RI) of the mobile phase caused by dissolved analytes, making them particularly useful for analytes lacking UV-absorbing chromophores like lipids.^11^

A common challenge in HPLC analysis of GOR products is peak overlap, particularly among glycerol, DHA, and FA. While this issue could potentially be addressed by using multiple columns with different properties,^13,14^ doing so comes at the cost of reduced method throughput. Chen et al. and Beltrán-Prieto et al. previously tackled the issue of elution peak overlap between glycerol and its oxidation products.^15,16^ However, difficulties arising specifically from the peak overlap between glycerol and DHA have continued to be reported by several researchers.^17,18^ Moreover, these studies did not consider FA,^15,16^ whose elution peak partially overlaps with those of glycerol and DHA, as discussed below. This issue of peak overlap could be one of the causes of the conflicting selectivity reported for BiVO_4_ photoanodes. Table 1 presents examples of GOR products along with their selectivity observed on undoped, nonco-catalyzed BiVO_4_ photoabsorbers in a pH 2 Na_2_SO_4_ solution. Despite employing the same material and electrolyte (except for the difference of the glycerol concentration), significant variations in the selectivity for GOR products were reported by different research groups. Although these discrepancies in selectivity might arise from varying reaction conditions, such as the applied potential and duration, or to differences in the BiVO_4_ preparation procedures, HPLC quantification errors due to peak overlap between glycerol, DHA, and FA cannot be ruled out. Furthermore, the complexity inherent in the analytical processes involved in identifying and quantifying GOR products is worth acknowledging, especially when a relatively large amount of unreacted glycerol is still present in the probed reaction environment.

**Table 1:** Examples of Glycerol Oxidation Reaction (GOR) Products on Undoped BiVO_4_ without Co-Catalysts in a pH 2 Na_2_SO_4_ Solution^a^

reference	electrolyte	reaction condition	1st major product	2nd major product	3rd major product
(19)	pH 2 Na_2_SO_4_ + 0.1 M glycerol	1.2 V_RHE_, 1 h	DHA (∼50%)	FA	GA
(20)	pH 2 Na_2_SO_4_ + 0.1 M glycerol	1.23 V_RHE_, 4 h	DHA (∼54%)	FA (∼35%)	GLAD (∼10%)
(21)	pH 2 Na_2_SO_4_ + 0.1 M glycerol	1.2 V_RHE_, 1 h	FA (∼65%)	DHA (∼20%)	GA (∼10%)
(22)	pH 2 Na_2_SO_4_ + 0.1 M glycerol	0.6 V_RHE_, 10 C	GCAD (40%)	DHA (24%)	FA (18%)
(23)	pH 2 Na_2_SO_4_ + 0.5 M glycerol	1.23 V_RHE_, 12 h	GCAD (43%)	DHA (22%)	GLAD (20%)
this work	pH 2 Na_2_SO_4_ + 0.5 M glycerol	0.6 V_RHE_, 13.9 h	GCAD (34.9%)	FA (26.4%)	DHA (24.3%)
this work	pH 2 Na_2_SO_4_ + 0.5 M glycerol	1.2 V_RHE_, 4.54 h	GCAD (34.7%)	FA (31.0%)	DHA (21.0%)

a The three major products and their corresponding selectivity are listed. Abbreviations: dihydroxyacetone (DHA), formic acid (FA), glyceric acid (GA), glycolaldehyde (GCAD), and glyceraldehyde (GLAD).

In this paper, we explore the challenges associated with analyzing GOR products using HPLC, particularly due to the overlapping peaks of glycerol, DHA and FA, and propose a method to address these challenges. First, we examine the optical properties of glycerol and GOR products to identify optimal wavelengths for spectrophotometric HPLC analysis. Second, we address the issue of peak overlap between glycerol and GOR products by proposing an algebraic solution utilizing multiple detectors—the RID and the VWD at various wavelengths. Third, we apply this method to analyze the GOR products produced by BiVO_4_ photoanodes. The key advantage of the protocol we present is that it can be applied using only HPLC with a single column, without requiring additional techniques such as proton nuclear magnetic resonance (^1^H NMR) spectroscopy. It would be useful to mention here that Higgins et al. recently published a paper on a quantification protocol for GOR products using ^1^H NMR.^24^ This, along with the work presented here, could serve as a roadmap for researchers studying glycerol valorization.

## Experimental Methods

### High-Performance Liquid Chromatography (HPLC)

An HPLC system (UltiMate 3000, Thermo Scientific), equipped with a HyperREZ XP H+ column (Thermo Scientific) with a length of 300 mm and a diameter of 7.7 mm, was employed in this study. The system featured a VWD (UltiMate 3000, Thermo Scientific) and a RID (RefractoMax 520, Thermo Scientific). A 5 mM H_2_SO_4_ aqueous solution served as the mobile phase, with the flow rate maintained at 0.5 mL min^–1^. The column temperature was consistently held at 60 °C. Reference chemicals for HPLC analysis included glycerol (>99%, Sigma-Aldrich), dihydroxyacetone (DHA, for synthesis, Sigma-Aldrich), formic acid (FA, 98–100%, Sigma-Aldrich), glycolic acid (GCA, 98%, Thermo Scientific), dl-glyceric acid (GA, ∼2 M in water, Chem Cruz), D-(+)-glyceraldehyde (GLAD, > 98%, Sigma-Aldrich), and glycolaldehyde dimer (GCAD, Sigma-Aldrich). These chemicals were dissolved in Milli-Q Type 1 water (18.2 MΩ) from a water purification system (Merck Millipore) for analysis. The measurements were conducted every 0.5 s (0.00833 min), starting from 0.00667 min. Therefore, the experimental uncertainty in the retention times reported in this work is approximately ±0.00417 min, calculated as half of the measurement interval (0.00833/2).

### UV Spectroscopy

UV spectroscopy was conducted with a Lambda 950 spectrophotometer (PerkinElmer). Glycerol and GOR product chemicals were dissolved in deionized water at a concentration of 0.1 M. The solutions were then placed in a quartz cuvette for analysis. Transmittance spectra were acquired using Milli-Q Type 1 water as reference. The absorbance was calculated from the transmittance using the following equation (%*T* represents transmittance expressed in percentage):
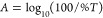
1

### Photoelectrochemical (PEC) Glycerol Oxidation

PEC measurements were performed using a potentiostat (VersaSTAT 3F, Princeton Applied Research). Nanoporous BiVO_4_ thin film photoanodes, characterized by a monoclinic scheelite crystalline structure, served as the model photoanode. The BiVO_4_ thin films were synthesized on fluorine-doped tin oxide (FTO) substrates through electrodeposition followed by postdeposition annealing, as reported elsewhere.^25^ Details of the synthesis method are provided in the Supporting Information (SI). The measurements employed a three-electrode system comprising the BiVO_4_ photoanode as the working electrode, an Ag/AgCl (saturated KCl) reference electrode (XR300, Radiometer Analytical), and a coiled platinum wire as the counter electrode. The applied potential relative to the Ag/AgCl (*V*_Ag/AgCl_) reference electrode was converted to the reversible hydrogen electrode (RHE) scale (V_RHE_) using the Nernst equation:
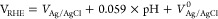
2where *V*^0^_Ag/AgCl_ is the standard potential of the reference electrode (0.197 V). PEC glycerol oxidation was carried out in a pH 2 Na_2_SO_4_ solution containing 0.5 M glycerol under AM1.5G 1-sun illumination provided by a solar simulator (WACOM WXS-50S-5H Class AAA) at an irradiance of 100 mW cm^–2^. Chronoamperometry (CA) was utilized, applying a constant potential of 0.6 V_RHE_ or 1.2 V_RHE_ until a total charge (*Q*_Total_) of 50 C cm^–2^ was achieved. Afterward, 1.5 mL of the electrolyte solution was collected for HPLC analysis. The relative selectivity (RS) for a product *i* (RS_*i*_) was calculated using the following equation:
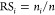
3where *n*_*i*_ represents the moles of product *i* produced (e.g., *n*_DHA_), and *n* signifies the total moles of all products. The Faradaic efficiency for product *i* (FE_*i*_) was calculated using the equation:
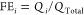
4where *Q*_*i*_ is the charge dedicated to the oxidation of glycerol into product *i* (e.g., Q_DHA_). The total Faradaic efficiency (FE_Total_) was calculated using the formula:
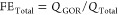
5where *Q*_Total_ is the total electric charge passed during the GOR, and *Q*_GOR_ is the electric charge specifically dedicated to the GOR. *Q*_GOR_ was calculated using the following formula:
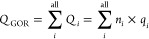
6where *q*_*i*_ represents the number of electrons consumed to produce one molecule of species *i* (e.g., *q*_DHA_). Based on the chemical reactions involved, *q*_*i*_ values for DHA (C_3_H_6_O_3_), GLAD (C_3_H_6_O_3_), GCAD (C_2_H_4_O_2_), and FA (CH_2_O_2_) are 2, 2, 4/3, and 8/3, respectively.
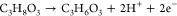
7
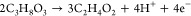
8
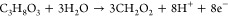
9

### Synthesis of Nanoporous BiVO_4_ Thin Films

Initially, 0.4 M potassium iodide (Santa Cruz Biotechnology) was dissolved in 50 mL of Milli-Q Type 1 water (18.2 MΩ). To this solution, 0.08 mL of nitric acid (∼70%, Honeywell) and 0.04 M bismuth(III) nitrate pentahydrate (98%, Acros Organics) were added. Concurrently, a 0.23 M p-benzoquinone (98%, Alfa Aesar) solution was prepared by dissolving it in 20 mL of high-purity ethanol (99.9%). The aqueous solution was then gradually introduced to the ethanolic solution, resulting in a dark red solution. BiOI nanosheet arrays were subsequently electrodeposited onto FTO substrates with a sheet resistance of 7 Ω sq^–1^ (Sigma-Aldrich). The electrodeposition setup comprised a working electrode (the FTO substrate), a coiled platinum wire counter electrode (0.5 mm diameter), and an Ag/AgCl (saturated KCl) reference electrode (XR300, Radiometer Analytical). Electrodeposition was conducted at a constant potential of −0.1 V_Ag/AgCl_ in the dark red solution until a total charge of 200 mC cm^–2^ was achieved, forming red-orange films. The films were then gently rinsed with Milli-Q Type 1 water. Following this, 50 μL cm^–2^ of a 0.2 M vanadyl acetylacetonate (Acros Organics) solution in dimethyl sulfoxide (99%, VWR Life Science) was drop-casted to the BiOI film. Annealing was performed at 450 °C for 2 h on a titanium hot plate (PZ28-3TD, Harry Gestigkeit GmbH), with a heating rate of 2 °C min^–1^, to facilitate the conversion to monoclinic BiVO_4_. The hot plate was then cooled to room temperature (25 °C) at a cooling rate of 2 °C min^–1^. Any excess V_2_O_5_ layer was removed by immersing the samples in a 1 M NaOH aqueous solution for 5 min.

### Structural and Morphological Characterization

X-ray diffraction (XRD) analysis was performed using an X-ray diffractometer (X’Pert, PANalytical). Cu Kα radiation, with a wavelength of 1.5406 Å, was utilized, and the incident angle of the X-ray was 2°. Scanning electron microscopy (SEM) analysis was conducted with a GeminiSEM 360 instrument (ZEISS).

## Results and Discussion

**Figure 1:**
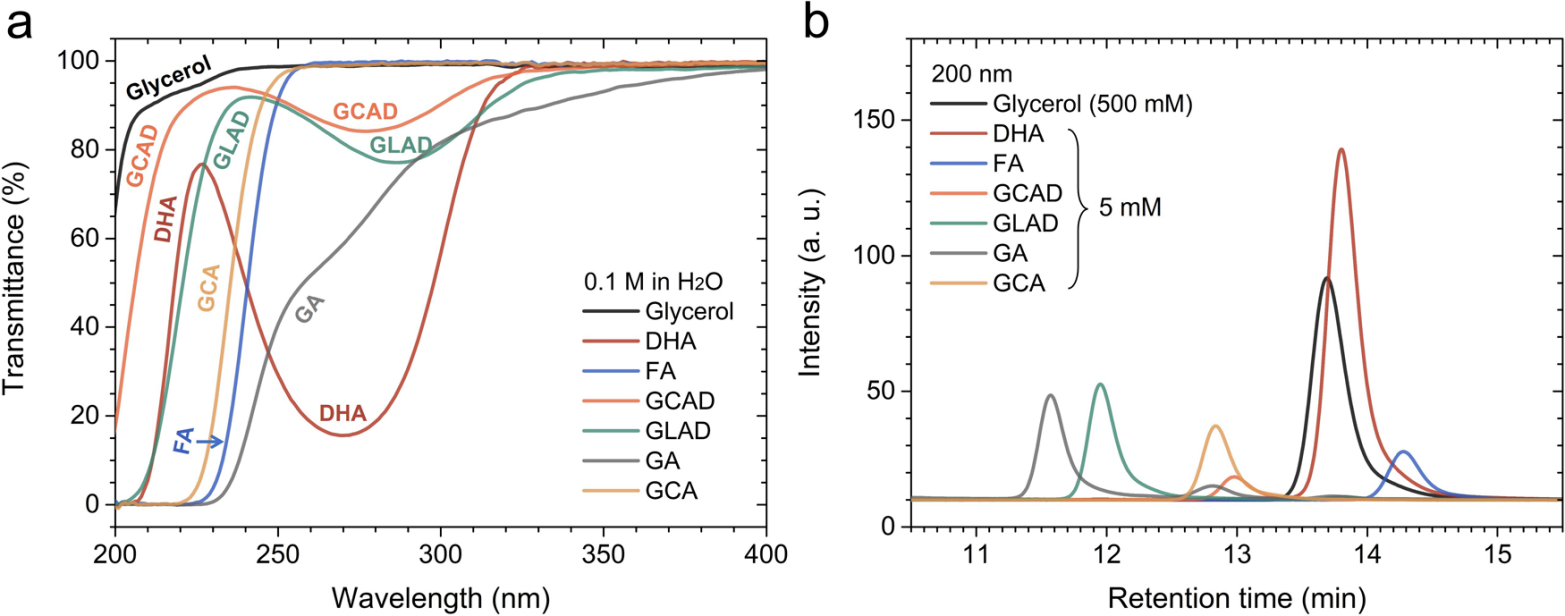
(a) Transmittance spectra of glycerol and glycerol oxidation reaction (GOR) products over a wavelength range from 200 to 400 nm. (b) High-performance liquid chromatography (HPLC) chromatograms of glycerol and GOR products, measured with a variable wavelength detector (VWD) at 200 nm. The chromatograms in (b) were individually obtained for each chemical and subsequently combined into a single graph for comparison. Abbreviations: DHA (dihydroxyacetone), FA (formic acid), GCAD (glycolaldehyde), GLAD (glyceraldehyde), GA (glyceric acid), GCA (glycolic acid).

Figure 1a displays the transmittance spectra for glycerol and various GOR products over a wavelength range from 200 to 400 nm. The absorbance spectra, derived from the transmittance spectra, are presented in Figure S1. These measurements were performed using 0.1 M aqueous solutions of the chemicals, with Milli-Q Type 1 water serving as the reference. All GOR products exhibit considerable absorbance at 200 nm, whereas the absorbance of glycerol at this wavelength range is relatively low. GLAD, GCAD, and particularly DHA show distinct absorption peaks between 270 and 280 nm, while GA shows an absorption shoulder in this region.

Based on the UV spectra, it was anticipated that glycerol and all GOR products could be identified and quantified using the VWD at 200 nm. Figure 1b displays chromatograms of glycerol (500 mM in H_2_O) and the GOR products (5 mM in H_2_O), measured with the VWD at 200 nm. These chromatograms were individually obtained for each chemical and subsequently combined into a single plot for comparison. The selection of concentration−500 mM for glycerol and 5 mM for GOR products–was based on typical conditions found in PEC glycerol oxidation studies.^26−30^ In these studies, glycerol concentrations are typically 100 mM or higher, reaching up to 2 M, whereas GOR product concentrations after continuous electrolysis (lasting 2–20 h) are only a few mM. To rationalize these numbers, we can consider a lab-scale setup involving a photoanode with an area of 1 cm^2^ for a 10-h PEC oxidation of glycerol at a constant current density of 5 mA cm^–2^ in a 0.1 L electrolyte solution. The total charge passed during the electrolysis (*Q*_Total_) is calculated to be 180 C, based on the following equation:
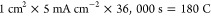
10

If the total charge *Q*_Total_ is solely due to GOR (i.e., negligible oxygen evolution reaction, OER) and DHA is produced with a selectivity of 50%, considering that the oxidation of glycerol to DHA requires two electrons per molecule, the resultant DHA concentration is calculated to be 4.66 mM using the following equation:
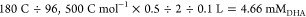
11

As shown in Figure 1b, an overlap exists among the elution peaks of the different GOR products; notably, the peaks of DHA and FA overlap with that of glycerol. Particularly, as will be discussed below, the peaks of glycerol and DHA appear as a single peak. Therefore, without sufficient caution, it is possible to incorrectly attribute the entire area of the peak to DHA alone, thereby overestimating the selectivity for DHA.

We aim to focus on the peak overlap of glycerol, DHA, and FA, as DHA and FA are among the most common GOR products, and their peak overlap with the glycerol reactant poses significant analytical challenges. Note that the overlapping peaks of GCA, GA and GCAD might also introduce complication when they are simultaneously present as GOR products, but the resolution of these peaks are beyond the scope of the current study. For calibration, chromatograms were collected for glycerol, DHA, and FA at several concentrations, as shown in Figure S2. We employed the VWD at two wavelengths—210 and 270 nm—and the RID. These chromatograms were integrated across a suitable retention time range, and the calculated mathematical areas were plotted as a function of concentration, showing the expected linear relationship (Figure S3).

**Figure 2:**
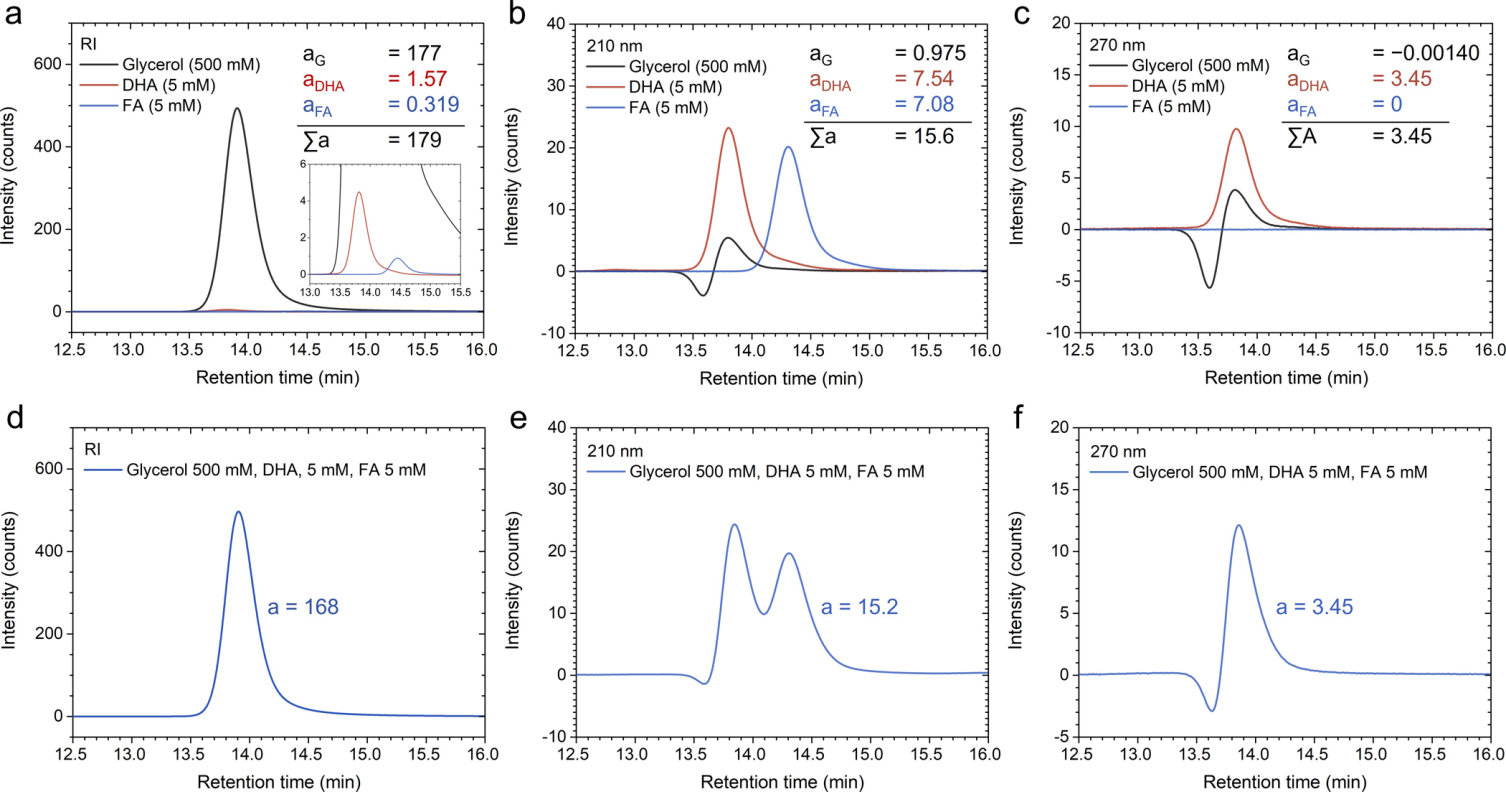
High-performance liquid chromatography (HPLC) chromatograms of 500 mM glycerol, 5 mM dihydroxyacetone (DHA), and 5 mM formic acid (FA) obtained using (a) the refractive index detector (RID), the variable wavelength detector (VWD) at wavelengths of (b) 210 nm, (c) 270 nm. (d–f) Chromatograms for a solution containing 500 mM glycerol, 5 mM DHA, and 5 mM FA, measured using (d) the RID, (e) the VWD at 210 nm, and (c) the VWD at 270 nm. The chromatograms in the plots from (a) to (c) were obtained individually for each chemical and subsequently compiled into a single plot for comparison.

Figure 2a–c show chromatograms for glycerol (500 mM), DHA (5 mM), and FA (5 mM) obtained using the RID and the VWD at 210 and 270 nm, respectively. As shown in Figure 2a, glycerol exhibits relatively high sensitivity to the RID compared to its low sensitivity to the VWD (Figures 1b and 2b,c). This indicates that glycerol causes a significant change in the RI of the mobile phase, making the RID effective for glycerol detection.

At 210 nm, as shown in Figure 2b, DHA and FA exhibit comparable sensitivity. Notably, glycerol displays a negative signal at a retention time of approximately 13.6 min. Such negative signals for glycerol are also observed when its concentration is significantly reduced (see Figure S4a,b) and when glycerol is mixed with DHA and FA (see Figure S4c,d). Negative signals in chromatograms obtained using light absorption detectors, such as the VWD, occur when an analyte absorbs less light than the mobile phase at a specific wavelength, resulting in a detector response lower than the baseline.^31^ Integration of the total signal for glycerol at 210 nm (e.g., from 13.2 to 14.5 min) yields an area linearly proportional to the concentration (see Figure S3d).

Negative signals for glycerol are also observed at 270 nm, as shown in Figure 2c. At 270 nm, integration of the total signal produces an area that is nearly zero, rendering linear fitting meaningless (see Figure S3g). Additionally, FA shows very low sensitivity at 270 nm. A 5 mM FA solution does not produce a quantifiable peak, and its calibration curve shows that FA’s sensitivity at 270 nm is only 0.2% of that of DHA.

Linearity is a key characteristic of HPLC chromatograms. First, the areas under the peaks are linearly proportional to the concentrations of the analyte, as demonstrated by the area–concentration graphs and corresponding linear fitting results for glycerol and GOR products (see Figure S3). This forms the basis for calculating the analyte’s concentration from the chromatogram. Second, the peak area of a mixture (e.g., a solution containing glycerol, DHA, and FA) is the linear sum of the peak areas of their individual chromatograms. This is demonstrated in Figure 2d–f, which show the chromatograms of a solution containing 500 mM glycerol, 5 mM DHA, and 5 mM FA, measured using the RID and the VWD at 210 and 270 nm, respectively. For instance, when the entire signal is integrated (e.g., from 13.2 to 15.5 min of retention time) in the RID chromatogram (Figure 2d), the result is 168 counts minute. This is comparable to the sum (179 counts minute) of the peak areas of 500 mM glycerol (177 counts minute), 5 mM DHA (1.57 counts minute), and 5 mM FA (0.319 counts minute) from their individual chromatograms (Figure 2a), corresponding to an error of 6.15%. At 210 nm (Figure 2b,e), the error is 2.56%, and at 270 nm (Figure 2c,f), the integration results are identical when three significant figures are considered.

These two aspects of linearity can be utilized alongside multiple detectors to quantify glycerol and GOR products without the need for peak deconvolution or multiple columns. Here, we present an algebraic method to quantify glycerol, DHA, and FA. To quantify *n* chemicals, *n* detectors are required (where each wavelength of the VWD is considered an independent detector). We note that this algebraic approach has been previously reported by researchers for the HPLC analysis of chemicals other than glycerol and GOR products.^32^

For calibration, chromatograms are collected at various concentrations. The peaks are integrated over an appropriate retention time range, and a graph can be plotted with the peak area (*a*) on the *y*-axis and the concentration (*c*) on the *x*-axis. Linear fitting of this graph yields the following equation:
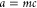
12where *m* is the slope. Since *a* must be zero when no analyte is present, the *y*-intercept is zero. We will denote the concentration of any analyte as *c*_analyte_ (e.g., *c*_FA_) and specify its calibration slope *m* for the detector used as *m*_Analyte_^Detector^ (e.g., *m*_DHA_^210^).

Consider a solution containing glycerol, DHA, and FA with concentrations *c*_G_, *c*_DHA_, and *c*_FA_, respectively, and its chromatogram obtained using three detectors: the RID, the VWD at 210 nm, and the VWD at 270 nm. Figure 2d–f can serve as examples. The integrated total signal (mathematical area) for a given detector will be denoted as *a*^Detector^ (e.g., *a*^RID^). For instance, in Figure 2d–f, this corresponds to the integration over the retention time range from 13.2 to 15.5 min.

Utilizing the two aspects of linearity discussed earlier, *a*^*RID*^, *a*^210^, and *a*^270^ can be expressed as follows (where “G” represents glycerol):
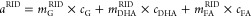
13
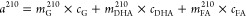
14
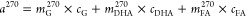
15

These three linear equations can be represented in matrix form as follows:
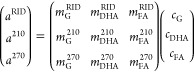
16
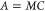
17

What we aim to determine is the matrix *C*, so we multiply both sides by the inverse of *M* (M^–1^):
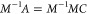
18
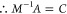
19

The matrix *M* (and consequently M^–1^) and matrix *A* can be determined through calibration and integration of the chromatograms, respectively. Therefore, with reliable calibration information and chromatograms obtained using at least three detectors, each component in a solution of glycerol, DHA, and FA can be quantified. It is worth noting that if *k* is the number of identified products, *k* different measurements (for instance using *k* different wavelengths) must be performed to obtain a matrix *M* with the maximum number of linearly independent rows and columns. In this case, the rank of the matrix *M* (rk(*M*)) equals *k* (rk(*M*) = *k*). Since we are searching for solutions within the entire , the Rouché–Capelli theorem guarantees a unique solution for the linear system described above.

The matrix *M* obtained from our calibration data shown in Figure S3 is as follows:
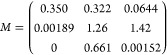
20

Its inverse *M*^–1^ is calculated as follows:
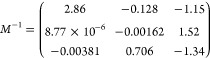
21

To validate the proposed methods, it was first applied to the chromatograms shown in Figure 2d–f. The calculated concentrations for glycerol, DHA, and FA are 475, 5.22, and 5.47 mM, respectively. These correspond to errors of 5.34, 4.43, and 9.40%, respectively. The proposed method was also applied to a solution where *c*_G_, *c*_DHA_, and *c*_FA_ were all 100 mM (see Figure S5 for the chromatograms of the solution). The calculated concentrations were 94.0, 99.6, and 105 mM, corresponding to errors of 6.0, 0.4, and 5.0%, respectively.

**Figure 3:**
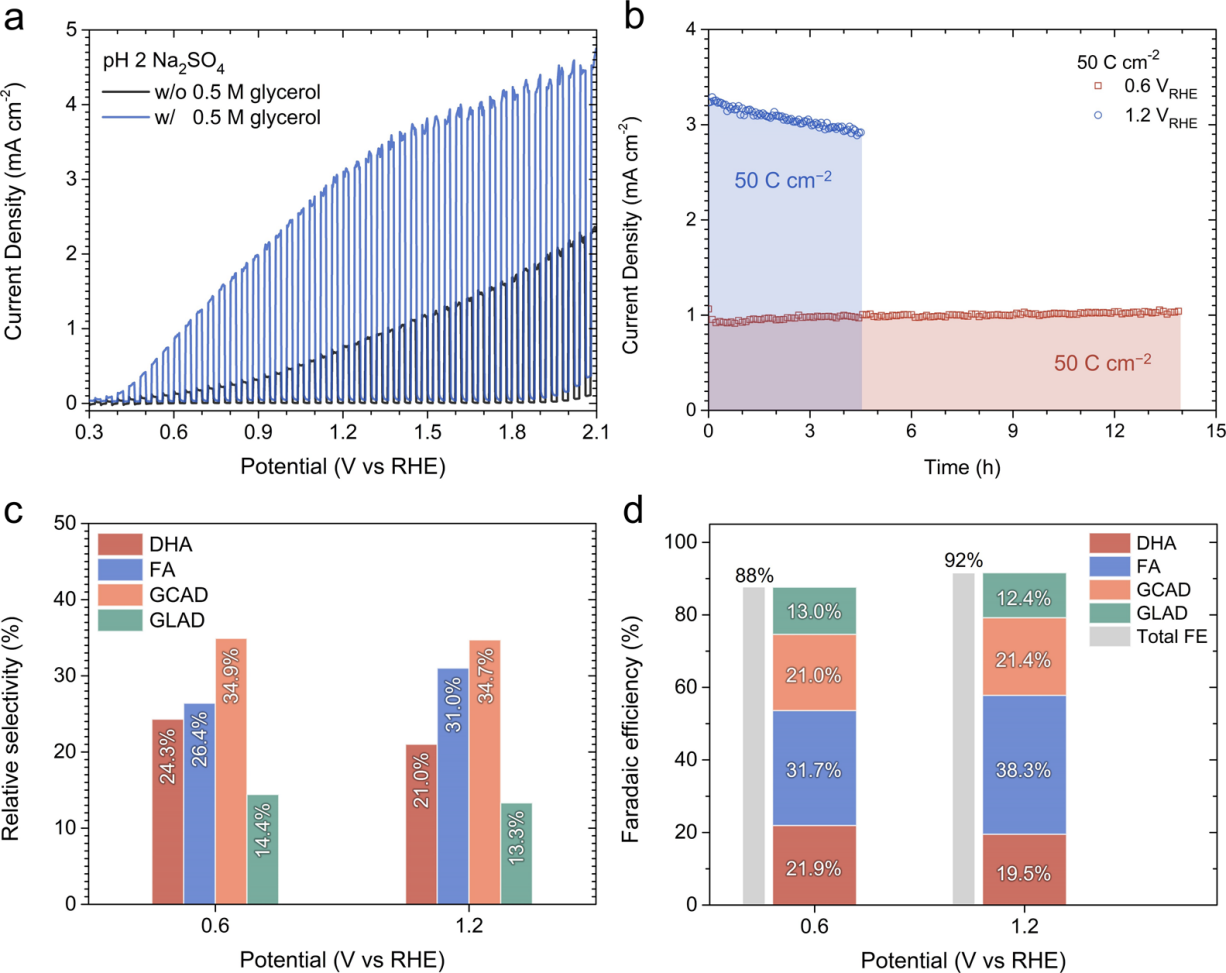
Analysis of glycerol oxidation reaction (GOR) products of undoped, nonco-catalyzed BiVO_4_ photoanodes. (a) Linear sweep voltammetry (LSV) curves measured in a pH 2 Na_2_SO_4_ solution, with and without 0.5 M glycerol. The measurements were performed at a scan rate of 20 mV s^–1^ under 1-Sun AM1.5G illumination. (b) Chronoamperometry (CA) curves recorded at +0.60 V_RHE_ and +1.20 V_RHE_ in a pH 2 Na_2_SO_4_ solution containing 0.5 M glycerol. The measurements proceeded until a total charge (*Q*_Total_) per unit area of 50 C cm^–2^ was achieved. (c) Relative selectivity (RS) and (d) Faradaic efficiency (FE) for various GOR products, including dihydroxyacetone (DHA), formic acid (FA), glycolaldehyde (GCAD), and glyceraldehyde (GLAD).

It should be acknowledged that relatively high errors were encountered in determining the concentrations of glycerol and FA. Notably, the estimated glycerol concentration was lower than expected, while the estimated FA concentration was higher than expected. As will be discussed in Figure 3, a similar trend of reduced glycerol concentration was observed in experiments with BiVO_4_ photoanodes. This discrepancy could be due to glycerol being converted into certain GOR products through spontaneous reactions, likely occurring in the relatively high temperature environment of the HPLC separation column. Specifically, the oxidation of glycerol to FA is characterized by a negative change in standard Gibbs free energy (Δ*G*^0^) of −0.714 kJ mol^–1^, suggesting the spontaneous nature of the reaction (detailed calculations are available in the SI, Supporting Note 1). This spontaneous oxidation of glycerol to FA could account for the relatively high errors in their quantification. Furthermore, as depicted in Figure S6a, a peak around 12.0 min of retention time, corresponding to approximately 0.1 mM of GLAD, was identified. This peak was also observed in the glycerol-only chromatograms (Figure S6b), albeit with lower intensity, indicating that glycerol can undergo a spontaneous, nonelectrochemical conversion to GLAD both in the absence and presence of DHA and FA. This could also have caused errors in the quantification. However, the GLAD peak at ∼12.0 min was not observed in the FA-only and DHA-only chromatograms, as shown in Figure S6c, indicating that the conversion of FA or DHA to GLAD did not occur.

It remains unclear under which conditions such spontaneous oxidation reactions occur. Possible favorable conditions could be established directly within the separating column in the HPLC setup, owing to the combined effect of relatively high temperature and presence of dissolved oxygen in the liquid stream. To investigate this, we purged the liquid sample with Ar gas (5N purity) for 30 min prior to the injection into the HPLC apparatus. The liquid samples were collected after the photoelectrolysis of glycerol using a BiVO_4_ photoanode (to be described in the following paragraph). As shown in Figure S7**,** the Ar purging did not alter the chromatogram, indicating that oxygen (or any other gases) present in the solution do not react with glycerol under the conditions of the HPLC measurement, which in our case included a column temperature of 60 °C. Hence, the oxidation of glycerol to GLAD and/or FA could spontaneously occur during the HPLC analysis due to the exposure to the column temperature (and eventual catalytic effects played by the sulfuric acid–base mobile phase), or during the storage of the liquid samples, even at temperatures below ambient conditions.

We have shown that by judicious use of different detectors during HPLC analysis, it is possible to resolve peak overlap among glycerol, DHA, and FA and quantify them within an acceptable margin of error. Next, we aim to apply the proposed method to the analysis of GOR products of BiVO_4_ photoanodes. We employed BiVO_4_ thin film photoanodes with a monoclinic phase, synthesized via electrodeposition on fluorine-doped tin oxide (FTO) substrates, as the model photoanode.^25^ Characterization results of the BiVO_4_ thin film photoanode, including X-ray diffraction (XRD), UV–vis spectroscopy, and digital and scanning electron microscopy (SEM) images, are presented in Figure S8. Initially, the GOR performance of BiVO_4_ photoanodes was assessed using linear sweep voltammetry (LSV), as shown in Figure 3a. A pH 2, 0.5 M Na_2_SO_4_ solution was selected as the electrolyte due to its prevalent use in studies on PEC glycerol oxidation with BiVO_4_ photoanodes, facilitating comparison. In this solution (without glycerol), our BiVO_4_ photoanode exhibited a photocurrent of 0.77 mA cm^–2^ at +1.23 V_RHE_. The addition of 0.5 M glycerol resulted in a notable increase in photocurrent across the entire potential range. For example, at +1.23 V_RHE_, the photocurrent increased to 3.10 mA cm^–2^. Chronoamperometry (CA) was utilized for PEC glycerol oxidation, conducted at potentials of +0.60 V_RHE_ and +1.20 V_RHE_ until a *Q*_Total_ per unit area of 50 C cm^–2^ was achieved (the exposed photoanode area was 0.5 cm^2^). The duration to reach 50 C cm^–2^ differed due to the photocurrent variation: 13.9 h was required at +0.60 V_RHE_, while only 4.54 h was needed at +1.20 V_RHE_ to obtain the same *Q*_Total_.

Following the CA measurements, liquid samples were collected for HPLC analysis. The liquid samples were stored in 1.5 mL vials sealed with rubber caps immediately after photoelectrolysis. HPLC measurements were conducted within 3 days of collection, and the samples were stored in a refrigerator in the meantime. The chromatograms, obtained using the VWD and the RID, are shown in Figure S9. By applying the proposed method, we determined the concentrations of the unreacted glycerol and oxidation products, enabling us to calculate their RS as depicted in Figure 3c. In the chromatogram at 200 nm (Figure S9b), the peaks at ∼12 and ∼13.1 min correspond to GLAD and GCAD, respectively, with their calibration details available in Figure S10. The absence of peak overlap enables a straightforward quantification of GLAD and GCAD from the 200 nm chromatogram. Notably, GCAD was identified as the most dominant GOR product at both potentials, with FA ranking as the second. The RS for GCAD was consistently around 35% at both potentials. DHA, ranking third among the GOR products, had an RS of 24.3% at +0.60 V_RHE_, which dropped to 21.0% at +1.20 V_RHE_. GLAD showed the lowest RS, approximately 13–14% for both potentials. We also calculated the FE for each GOR product and the FE_Total_, as shown in Figure 3d. The FE_Total_ was about 90% for both potentials, with the remaining 10% potentially due to photocorrosion or GOR products undetectable by HPLC, such as CO_2_. Given that the transformation of glycerol to FA involves the highest electron consumption per molecule of FA (8/3), FE_FA_ was the highest at both potentials. Despite GCAD being the most significant in terms of RS, FE_GCAD_ was lower because the glycerol to GCAD oxidation process requires only 4/3 electrons (DHA and GLAD oxidation require two electrons per molecule).

**Figure 4:**
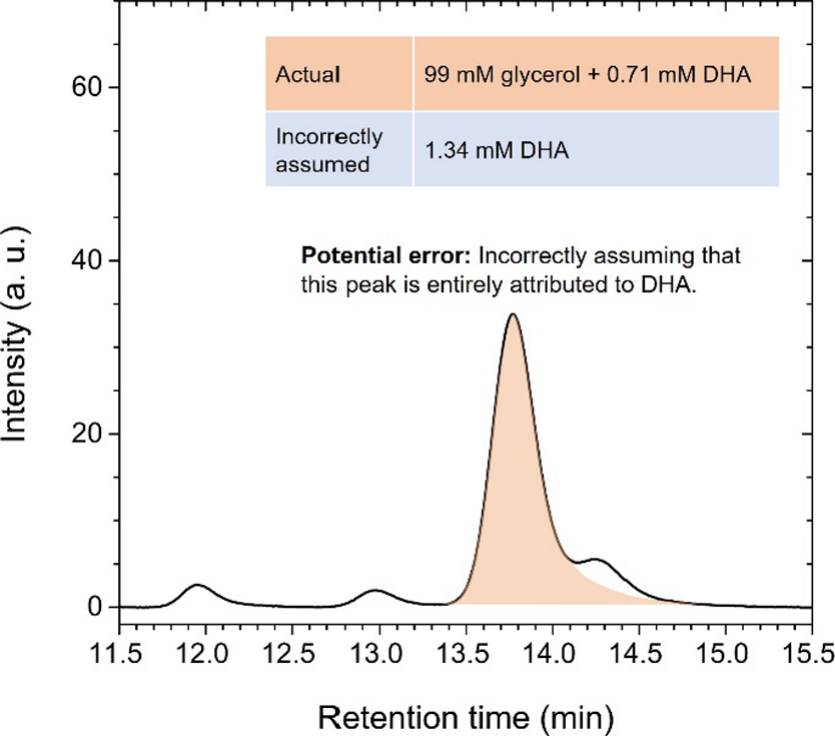
Chromatogram of a 0.5 M Na_2_SO_4_ solution (pH = 2) containing 99 mM glycerol, 0.71 mM DHA, 0.77 mM FA, 1.02 mM GCAD, and 0.42 mM GLAD, measured at 200 nm. One potential mistake is assuming that the peak at ∼13.8 min is solely due to DHA, when in fact the peaks of glycerol and DHA are overlapping.

Before concluding, it is important to explain how misleading results can arise in GOR product analysis. A potential error lies in overlooking the peak overlap between glycerol and DHA and mistakenly assuming that the peak at ∼13.8 min is solely due to DHA. To illustrate this, let us assume that GOR products were produced with the same selectivity as in the case of 0.6 V_RHE_ shown in Figure 3c, but with an initial glycerol concentration of 100 mM. Based on this assumption, we prepared a 0.5 M Na_2_SO_4_ solution (pH = 2) containing 99 mM glycerol, 0.71 mM DHA, 0.77 mM FA, 1.02 mM GCAD, and 0.42 mM GLAD. The chromatogram of this solution, obtained at 200 nm, is shown in Figure 4. Using our method, the concentrations of each solute were calculated to be as follows (with error percentages in parentheses): 96 mM for glycerol (3%), 0.73 mM for DHA (3%), 0.80 mM for FA (4%), 0.96 mM for GCAD (6%), and 0.39 mM for GLAD (8%). This results in a FE_Total_ of 87%. If we assume that the peak at ∼13.8 min is solely attributed to DHA, the DHA concentration would be calculated as 1.34 mM. Nevertheless, if the GCAD concentrations were correctly calculated, it would immediately be evident that an error occurred in the quantification, as the FE_Total_ would exceed 100%.

It is worth noting that in studies reporting DHA as the primary product, GCAD has not been identified as a product (Table 1). If GCAD is overlooked, the RS values for DHA, FA, and GLAD would be calculated as 53.0, 30.4, and 16.6%, respectively, closely matching the RS values reported in those studies (Table 1). Additionally, the FE_Total_ would be calculated as 86%, which might appear reasonable. A similar issue could arise if GCAD is misidentified as GCA. GCAD and GCA show peaks at similar retention times (Figure 1b). However, due to its higher UV absorption (Figure 1a), GCA is calculated to have a lower concentration than GCAD, even when the peak areas are the same. If the peak at ∼13.8 min is fully attributed to DHA and GCAD is misidentified as GCA, the RS values for DHA, FA, GLAD, and GCA would be calculated as 47.0, 27.0, 14.7, and 11.2%, respectively. The FE_Total_ would then be calculated as 96%, still within an acceptable range. Such plausible values make it even easier to overlook the peak overlap between glycerol and DHA. Therefore, special attention should be paid when quantifying GOR products.

## Conclusions

In this study, we addressed the issue of peak overlap between glycerol, DHA, and FA and proposed a resolution. Initially, the UV absorption characteristics of glycerol and various GOR products in the aqueous phase were investigated. All chemicals demonstrated significant absorption at 200 nm, while DHA, GCAD, and GLAD exhibited absorption peaks around 270 nm. Subsequently, an algebraic quantification method was developed, leveraging the linearity property of HPLC chromatograms and multiple detectors. Our protocol successfully quantified glycerol, DHA, and FA within an acceptable error margin (less than 10%) despite overlapping peaks. However, relatively larger errors, up to 10%, were observed for glycerol and FA, possibly due to spontaneous reactions of glycerol. Finally, the proposed method was applied to analyze GOR products generated by BiVO_4_ photoanodes in a pH 2 Na_2_SO_4_ solution, with GCAD identified as the most dominant product.

## Data Availability

The data supporting this study are available at https://drive.google.com/file/d/1cLTtRENvw05Ma-j9CCiaRAS0_RBQ0OhR/view?usp=drive_link.

## Supporting Information Available

Calculation of the standard reduction potential (*E*^0^); standard Gibbs free energy of formation (Δ_f_*G*^0^) for hydrogen, water, glycerol, and FA; absorbance spectra of glycerol and GOR products; chromatograms of glycerol, DHA, and FA for calibration; peak area vs concentration graphs for glycerol, DHA, and FA; HPLC chromatograms of aqueous solutions containing 5 mM or 50 mM glycerol, 5 mM DHA, and 5 mM FA; chromatograms for a solution containing 100 mM glycerol, 100 mM DHA, and 100 mM FA; chromatograms for a solution containing 500 mM glycerol, 5 mM DHA, and 5 mM FA; chromatograms of electrolyte solutions after the photoelectrolysis of glycerol, with and without Ar gas purging; characterization results for the BiVO_4_ thin film photoanode; chromatograms obtained after CA tests at 0.6 V_RHE_ or 1.2 V_RHE_; and calibration data for GCAD and GLAD (PDF)
