# Supplementary material for: Resolving Peak Overlap in HPLC Analysis of Glycerol Oxidation Products by Utilizing Various Detectors: Application to BiVO4 Photoanodes
Source: ACS Omega. 2025 Mar 18;10(12):11786–95. doi: 10.1021/acsomega.4c07497 (PMC11966247; doi:10.1021/acsomega.4c07497)
Supplement: Supplementary file 1 — ao4c07497_si_001.pdf [file ao4c07497_si_001.pdf]

Resolving Peak Overlap in HPLC Analysis of Glycerol Oxidation Products by Utilizing Various Detectors: Application to $BiVO_4$ Photoanodes

Heejung Kong[1, 2, *], *Siddharth Gupta*[3, 4], *Matthew T. Mayer*[3, 4], *Eva Ng*[5], *Camilo A. Mesa*[5], *Sixto Giménez*[5], *Fatwa F. Abdi*[6], *Roel van de Krol*[1, 2], *and Marco Favaro*[1, *]

[1] Institute for Solar Fuels, Helmholtz-Zentrum Berlin für Materialien und Energie GmbH, Hahn-Meitner-Platz 1, 14109 Berlin, Germany

[2] Institute for Chemistry, Faculty Ⅱ – Mathematics and Natural Sciences, Technische Universität Berlin, Straße des 17. Juni 124, 10623 Berlin, Germany

[3] Electrochemical Conversion, Helmholtz-Zentrum Berlin für Materialien und Energie GmbH, Hahn-Meitner-Platz 1, 14109 Berlin, Germany

[4] Institute of Chemistry and Biochemistry, Department of Biology, Chemistry, and Pharmacy, Freie Universität Berlin, 14195 Berlin, Germany

[5] Institute of Advanced Materials, Universitat Jaume I, Avinguda de Vicent Sos Baynat, s/n, 12006 Castelló de la Plana, Spain

[6] School of Energy and Environment, City University of Hong Kong, 83 Tat Chee Avenue, Hong Kong SAR, China

[*] Authors to whom correspondence should be addressed.

H. Kong: heejung.kong@helmholtz-berlin.de

M. Favaro: marco.favaro@helmholtz-berlin.de

Supplementary Note 1: Calculation of the standard reduction potential (E^0)

The standard Gibbs free energy of formation ($\Delta_f G^0$) for water, hydrogen, and glycerol, as reported in the literature, is summarized in **Table S1**. The standard enthalpy of formation ($\Delta_f H^0$) and standard molar entropy (S^0) for formic acid (FA) were found to be −425 kJ mol^{-1} and 132 J K^{-1} mol^{-1}, respectively. [1, 2] Using these values, the $\Delta_f G^0$ of FA was calculated to be −464 kJ mol^{-1} employing the formula:

$$\Delta_f G^0 = \Delta_f H^0 - TS^0$$

where T (temperature) was set to room temperature (298.15 K). In the context of the oxidation reaction of glycerol to FA ($C_3H_8O_3 + 3H_2O + 8e^- \rightarrow 3CH_2O_2 + 4H_2$), the change in standard Gibbs free energy (ΔG^0) for the reaction was calculated to be −0.714 kJ mol^{-1}. From this value, the standard reduction potential (E^0) of the reaction was calculated to be −0.925 mV, using the Nernst equation:

$$\Delta G^0 = -nFE^0$$

where n is the number of moles of electrons transferred in the reaction (8), and F is Faraday constant (96485 C mol^{-1}).

Table S1. Standard Gibbs free energy of formation ($\Delta_f G^0$) of hydrogen, water, glycerol, and formic acid (FA).

Chemical	$\Delta_f G^0$ (kJ mol^{-1})
Hydrogen (*g*)	0[3]
Water (*l*)	−237[3]
Glycerol	−680[4]
FA	−464

Figures for Supporting Information

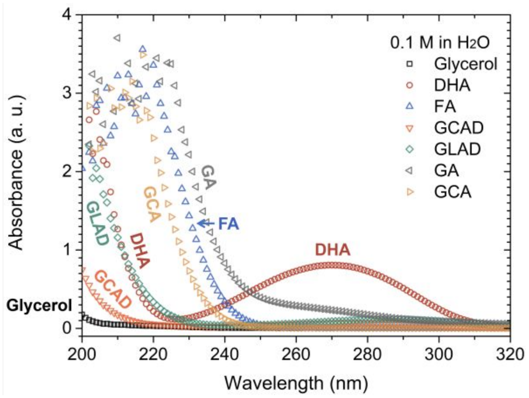

Figure S1. Absorbance spectra of glycerol and glycerol oxidation reaction (GOR) products.

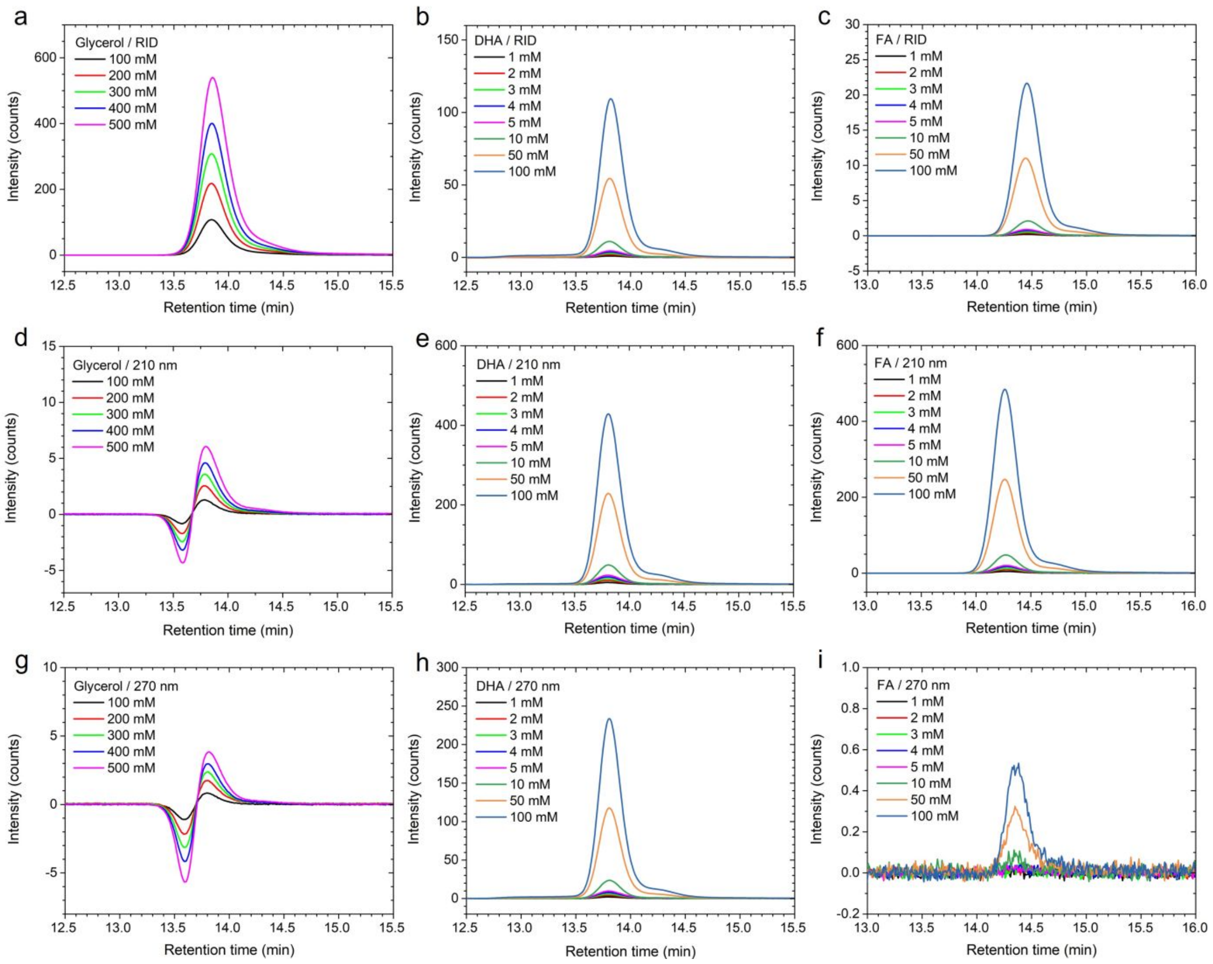

Figure S2. Calibration data obtained using **(a)**–**(c)** the refractive index detector (RID), **(d)**–**(f)** the variable wavelength detector (VWD) at 210 nm, and **(g)**–**(i)** the VWD at 270 nm for glycerol (**(a)**, **(d)**, and **(g)**), dihydroxyacetone (DHA) (**(b)**, **(e)**, and **(h)**), and formic acid (FA) (**(c)**, **(f)**, and **(i)**).

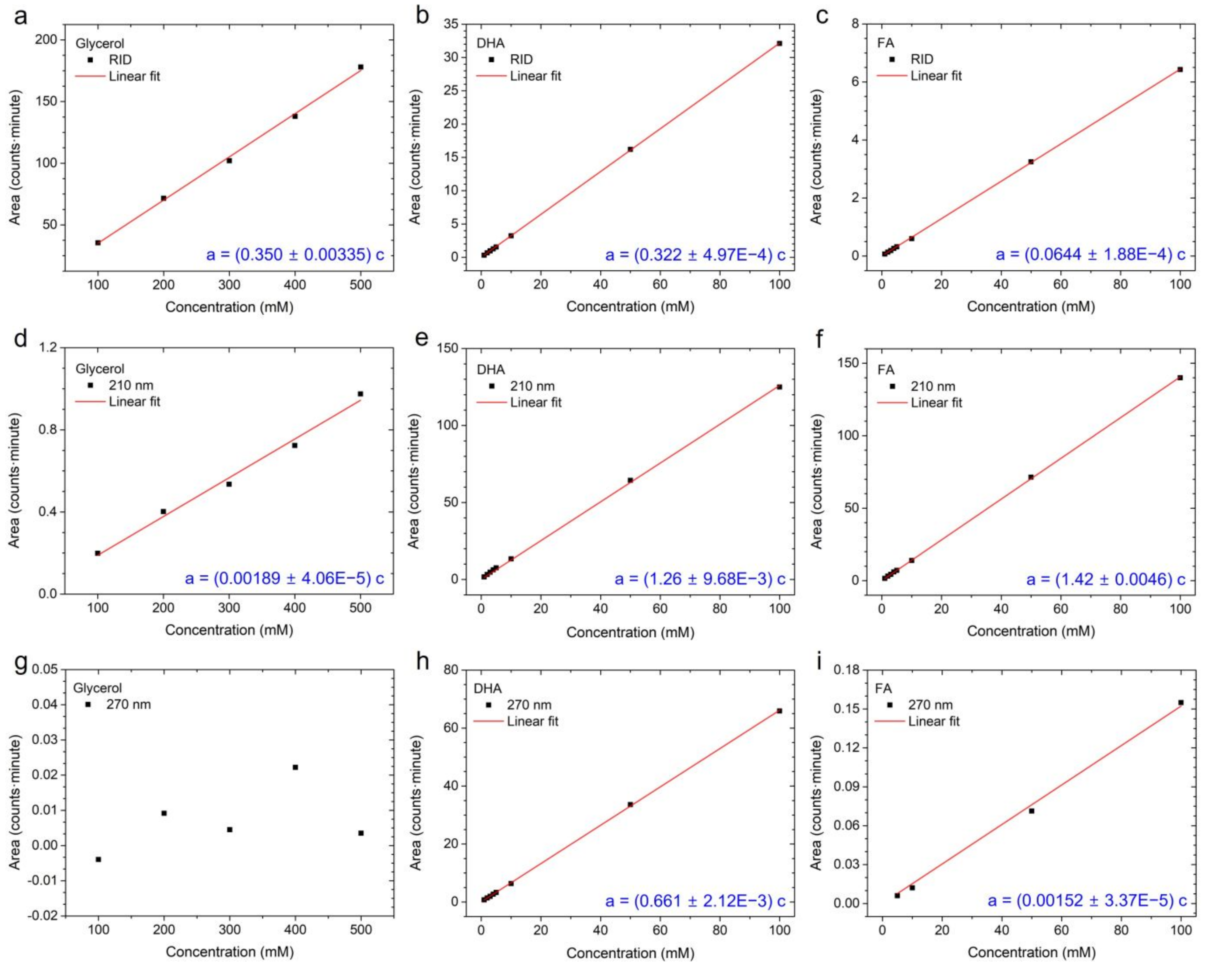

Figure S3. Peak area vs. concentration graphs for glycerol (**(a)**, **(d)**, and **(g)**), dihydroxyacetone (DHA) (**(b)**, **(e)**, and **(h)**), and formic acid (FA) (**(c)**, **(f)**, and **(i)**), obtained using **(a)**−**(c)** the refractive index detector (RID), **(d)**−**(f)** the variable wavelength detector (VWD) at 210 nm, and **(g)**−**(i)** the VWD at 270 nm.

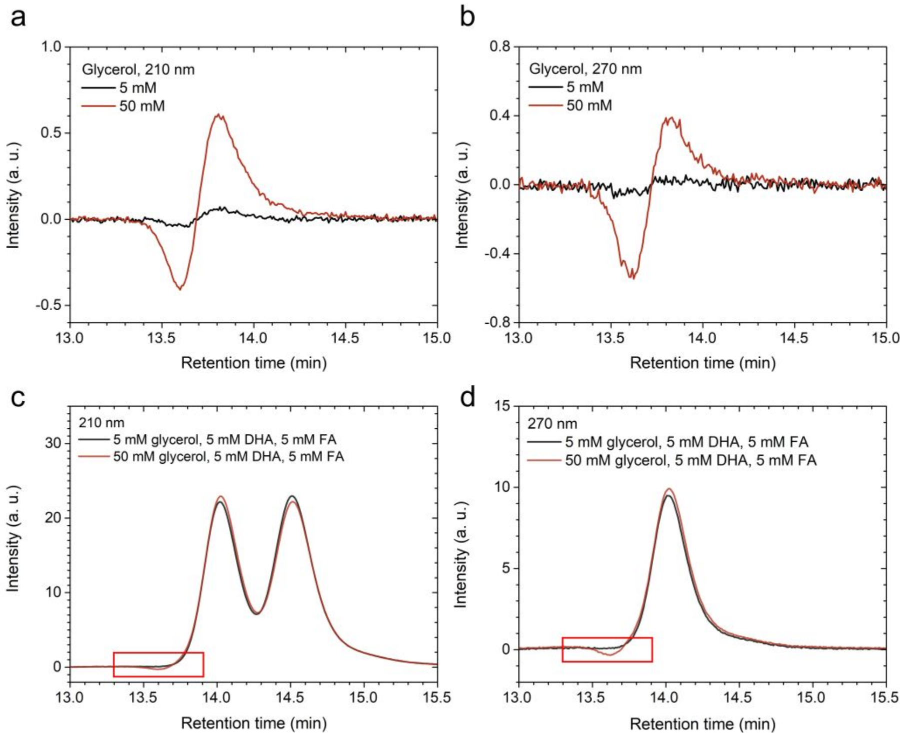

Figure S4. High-performance liquid chromatography (HPLC) chromatograms of aqueous solutions containing 5 mM or 50 mM glycerol measured using the variable wavelength detector (VWD) at **(a)** 210 nm and **(b)** 270 nm. Chromatograms of aqueous solutions containing glycerol (5 or 50 mM), dihydroxyacetone (DHA, 5 mM), and formic acid (FA, 5 mM) measured using the VWD at **(c)** 210 nm and **(d)** 270 nm.

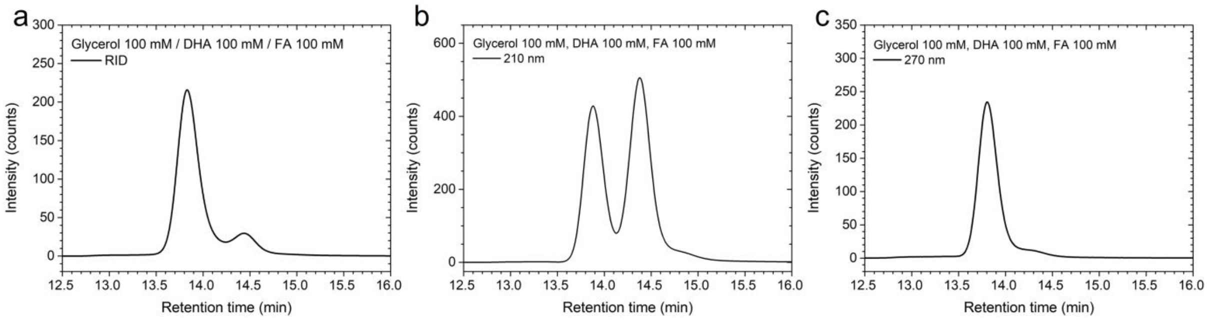

Figure S5. Chromatograms for a solution containing 100 mM glycerol, 100 mM dihydroxyacetone (DHA), and 100 mM formic acid (FA), measured using **(a)** the refractive index detector (RID), **(b)** the variable wavelength detector (VWD) at 210 nm, and **(c)** the VWD at 270 nm.

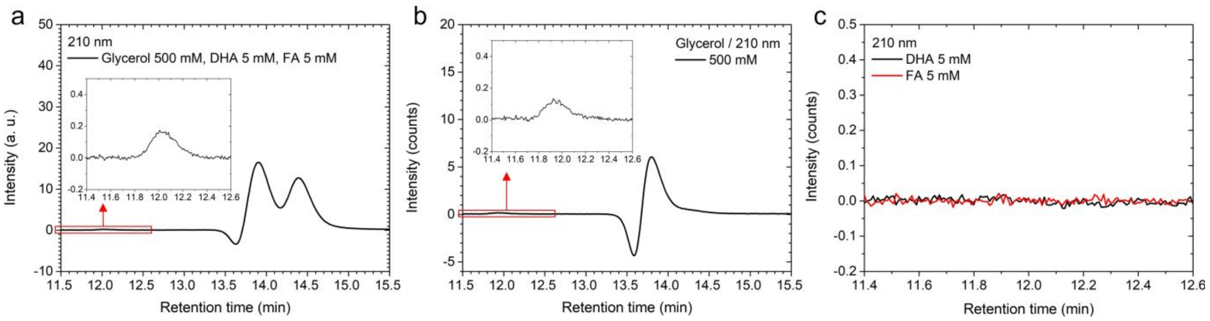

Figure S6. Chromatograms obtained using the variable wavelength detector (VWD) at 210 nm for **(a)** a solution containing 500 mM glycerol, 5 mM dihydroxyacetone (DHA), and 5 mM formic acid (FA), **(b)** a solution containing 500 mM glycerol, and **(c)** a solution of 5 mM DHA and a solution of 5 mM FA.

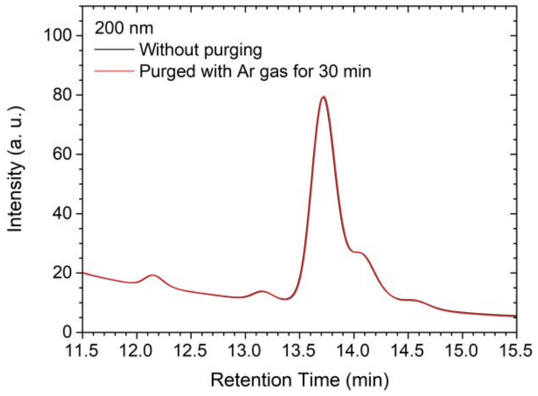

Figure S7. Chromatograms of electrolyte solutions after the photoelectrolysis of glycerol, without Ar gas purging (black curve) and with Ar gas purging for 30 minutes (red curve). The photoelectrolysis was conducted in a pH 2 0.5 M Na_2SO_4 solution using a nanoporous $BiVO_4$ photoanode under AM1.5G 1-Sun illumination (characterization results for the $BiVO_4$ photoanode are shown in **Figure S8**). A constant potential of +1.20 V_{RHE} was applied until a charge of 50 C cm^{-2} was achieved, followed by the injection of Ar gas into the solution for 30 minutes.

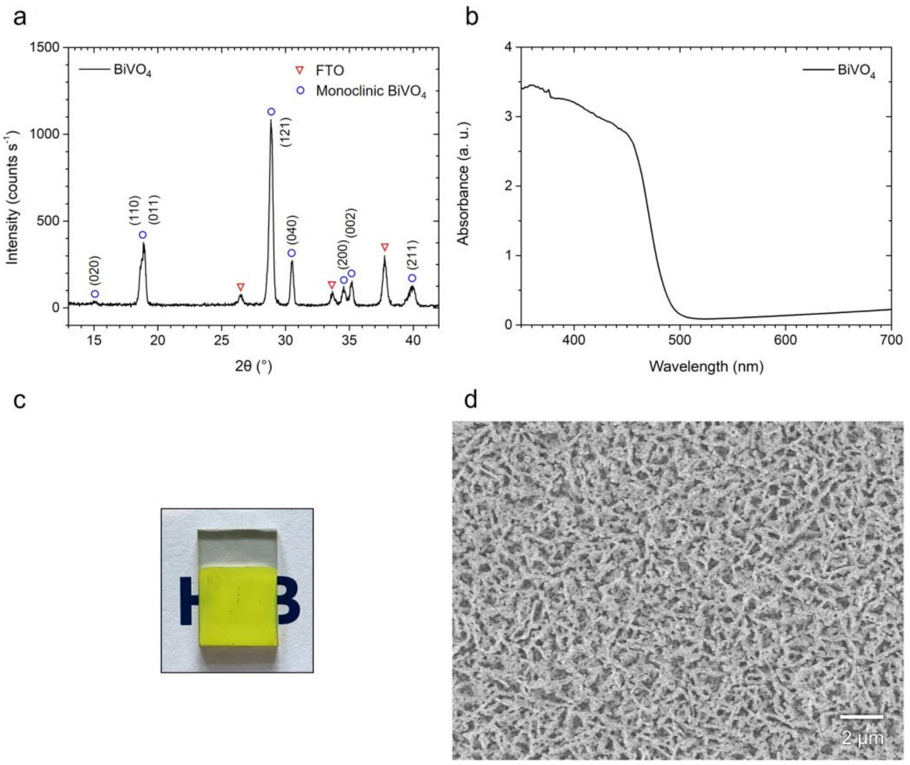

Figure S8. Characterization results for the $BiVO_4$ thin film photoanode. **(a)** X-ray diffraction (XRD) diffractogram, **(b)** UV-vis absorbance spectrum, **(c)** digital photograph, and **(d)** scanning electron microscopy (SEM) image of the $BiVO_4$ thin film.

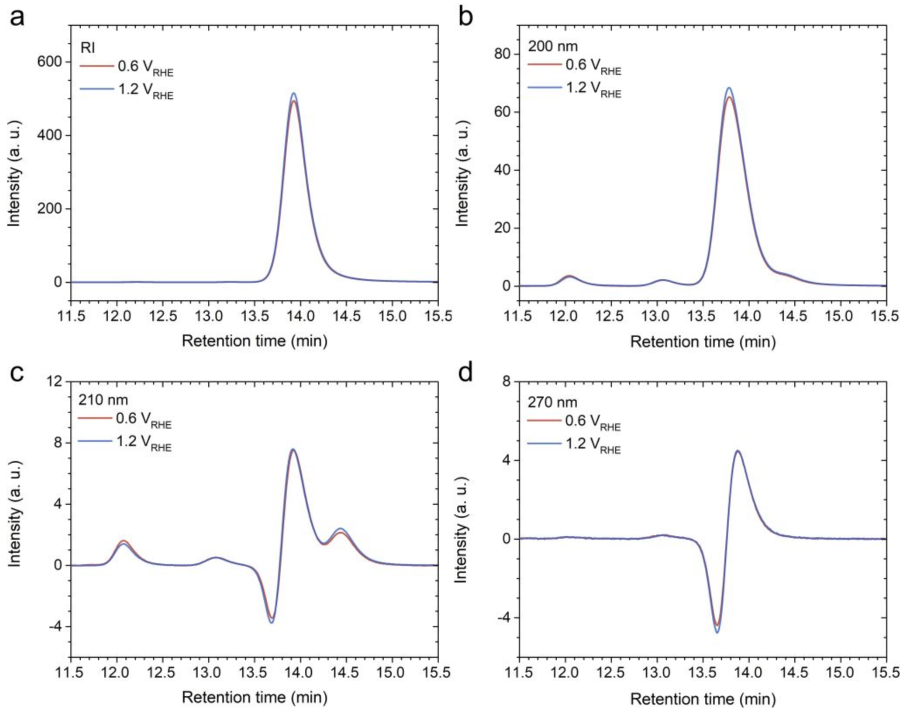

Figure S9. Application with $BiVO_4$ photoanodes. Chromatograms obtained using **(a)** the refractive index detector (RID) and the variable wavelength detector (VWD) at **(b)** 200 nm, **(c)** 210 nm, and **(d)** 270 nm. Liquid samples were collected following chronoamperometry (CA) at 0.6 V_{RHE} or 1.2 V_{RHE} and subjected to HPLC analysis. CA was continued until a total charge (Q_{Total}) of 50 C cm^{-2} (exposed area = 0.5 cm^2) was reached at each potential.

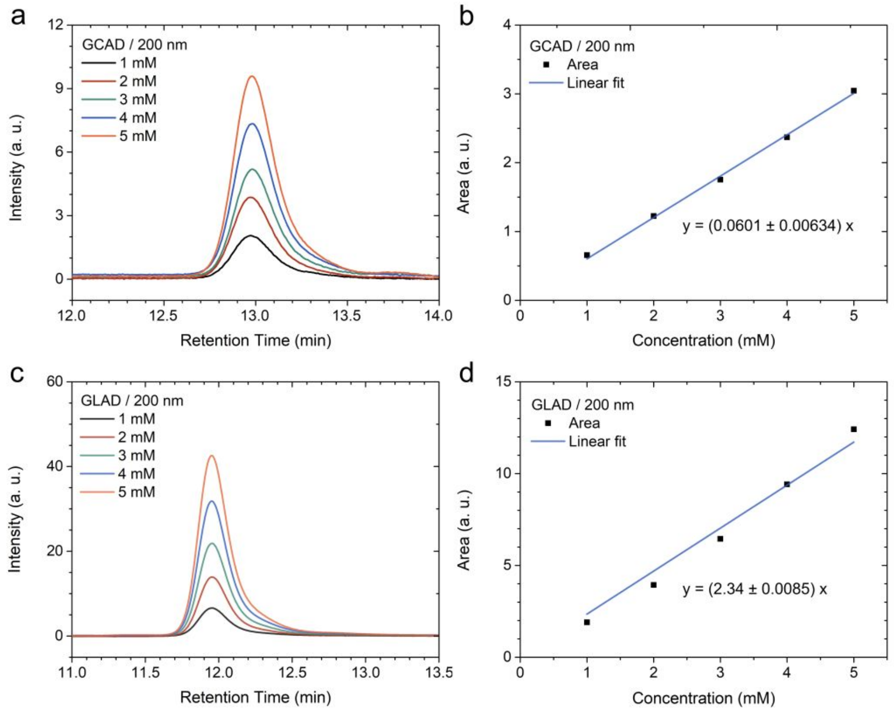

Figure S10. Calibration data for glycolaldehyde (GCAD) and glyceraldehyde (GLAD). **(a)**, **(c)** chromatograms obtained using the variable wavelength detector (VWD) at 200 nm for **(a)** GCAD and **(c)** GLAD. **(b), (d)** Peak area plotted as a function of concentration for **(b)** GCAD and **(d)** GLAD.

References for the Supporting Information

(1) Stout, J. W.; Fisher, L. H. The Entropy of Formic Acid. The Heat Capacity from 15 to 300°K. Heats of Fusion and Vaporization. *J. Chem. Phys.* **1941**, *9* (2), 163-168, DOI: 10.1063/1.1750869

(2) Guthrie, J. P. Hydration of carboxamides. Evaluation of the free energy change for addition of water to acetamide and formamide derivatives. *J. Am. Chem. Soc.* **1974**, *96* (11), 3608-3615, DOI: 10.1021/ja00818a039

(3) Cox, J. D.; Wagman, D. D.; Medvedev, V. A. *CODATA Key Values for Thermodynamics*; Hemisphere Publishing Corp., 1989.

(4) Bastos, M.; Nilsson, S.-O.; Ribeiro da Silva, M. D. M. C.; Ribeiro da Silva, M. A. V.; Wadsö, I. Thermodynamic properties of glycerol enthalpies of combustion and vaporization and the heat capacity at 298.15 K. Enthalpies of solution in water at 288.15, 298.15, and 308.15 K. *J. Chem. Thermodyn.* **1988**, *20* (11), 1353-1359, DOI: 10.1016/0021-9614(88)90173-5
